# Dietary 5-hydroxytryptophan supplementation improves growth performance and intestinal health of weaned piglets

**DOI:** 10.1186/s40813-024-00412-7

**Published:** 2024-12-20

**Authors:** Yinzhao Xia, Xie Peng, Jiani Mao, Ju Luo, Huifeng Li, Dengjun Ma, Zhenguo Yang

**Affiliations:** Laboratory for Bio-Feed and Molecular Nutrition, College of Animal Science and Technology, Southwest University, Chongqing, 400715 China

**Keywords:** 5-hydroxytryptophan, Weaned piglets, Growth performance, Intestinal health

## Abstract

This study investigated the effects of dietary 5-hydroxytryptophan (5-HTP) supplementation on growth performance, apparent total tract digestibility (ATTD), blood profile, intestinal morphology, transcriptomics, and microbial composition in weaned piglets. A total of twenty-four 28-day-old weaned piglets (Landrace × Large Yorkshire, 8.28 ± 1.09 kg) were randomly divided into 3 dietary treatments with 8 replicates. The dietary treatments include basal diet (CON), CON diet containing 250 or 500 mg/kg 5-HTP. The results revealed that supplementation with 250 mg/kg 5-HTP significantly increased (*P* < 0.05) the average daily gain (ADG) and resulted in a lower (*P* < 0.05) feed conversion ratio (FCR), while also decreased (*P* < 0.05) the diarrhea rate compared to the CON group. The ATTD of crude protein (CP) was lower in the 500 mg/kg group (*P* < 0.05) compared with the 250 mg/kg group. Furthermore, supplementation with 5-HTP led to significantly increased (*P* < 0.05) plasma albumin (ALB) and total protein (TP). In addition, supplementation with 5-HTP, particularly in the 250 mg/kg group, significantly increased (*P* < 0.05) serum serotonin (5-HT), growth hormone (GH) and insulin-like growth factor 1 (IGF-1) levels, and improved the ratio of villus height to crypt depth in the jejunum and ileum. The transcriptomic analysis revealed that the majority of differentially expressed genes (DEGs) induced by 5-HTP were related to digestion and immunity in the ileum, and 5-HTP enhanced (*P* < 0.05) intestinal glucose transporter 2 (*GLUT2*), solute carrier family 1 member 1 (*SLC1A1*) and solute carrier family 7 member 7 (*SLC7A7*) mRNA expression in weaned piglets. Furthermore, supplementation with 250 mg/kg 5-HTP increased (*P* < 0.05) abundance of *Firmicutes*, *Actinobacteriota*, *Lachnospiracea*, *Ruminococcaceae* and *Megasphaera* and decreased (*P* < 0.05) abundance of *Spirochaetes* and *Treponema*. Collectively, the study demonstrated that 5-HTP supplementation, particularly at 250 mg/kg, positively impacted growth performance, gut health, and microbiome composition in weaned piglets. These findings suggest the potential of using 5-HTP as a dietary supplement to enhance the health and productivity of weaned piglets.

## Introduction

To maximize reproductive potential throughout the lifespan of sows and minimize disease transmission from sows to piglets, early weaning has become a fundamental practice in modern intensive swine production [1]. During the early weaning period, the piglets have not yet formed well-performing digestive and immune systems, associated with the influence of multiple stress including physiological, nutritional, and environmental pressure, which severely affect the growth performance, physiological and metabolic activities, and cause disorders in the intestinal microbiota [2, 3]. Thus, it is more important to find functional ingredients to alleviate weaning stress during the post-weaning period.

5-HTP is a metabolite of tryptophan and serves as a direct precursor to 5-hydroxytryptamine (5-HT) [4, 5]. Previous studies suggested that dietary tryptophan (Trp) could increase growth performance through improving antioxidant capacity, immunity and gut microbiota in piglets [6–8]. Moreover, 5-HT is involved in regulating animal feeding, endocrine, intestinal flora and homeostasis as a monoamine neurotransmitter [9–13]. It has been widely reported that 5-HTP acts as a hydroxyl radical scavenger to resist oxidative damage in *vitro* experiment [14–16]. Meanwhile, 5-HTP has been shown to greater body 5-HT availability than use itself [17]. Dietary supplementation with 5-HTP improves intestinal immune function in broilers [18], and 5-HTP oral administration restores depression-induced gut microbiota dysbiosis in mice [19]. However, few studies have been conducted to access the impact of dietary 5-HTP on the ability to alleviate weaning stress in piglets. Here, we hypothesized that dietary 5-HTP would promote growth performance through improving intestinal health in weaned piglets. Therefore, this study was conducted to assess the effects of dietary 5-HTP on growth performance, nutrient digestibility, blood biochemistry, intestinal morphology and microbiota of weaned pigs.

## Materials and methods

### Materials and experimental design

According to the Institutional Animal Care and Use Committee of Southwest University, Chongqing, China (NO. IACUC-20220727-01), twenty-four 28-day-old weaned piglets (Landrace × Large Yorkshire, 8.28 ± 1.09 kg) were randomly divided into 3 dietary treatments with 8 replicates. Dietary treatments include basal diet (CON), CON diet containing 250 (250 mg/kg) or 500 (500 mg/kg) 5-HTP (Baoliruihe, Biotechnological Co LTD., Hebei, China). The dosages of 5-HTP were determined based on previous studies [20, 21]. The basal diet was formulated according to NRC (2012) recommendation. The composition and nutrients of the basal diet is detailed in Table 1. During the 35-day experimental period, piglets were provided with free access to both diets and water.

**Table 1:** Ingredients and nutrient content of the basal diet (as-fed basis)

Ingredients (%)		Nuritional level^b^	
Corn	60.01	DE (MJ/Kg)	14.82
Fermented Soybean meal	12.12	CP (%)	18.40
Puffed soybeans	8.11	CF (%)	2.44
Whey	9.67	Lys (%)	1.31
Fat powder	1.60	Met (%)	0.38
Fish meal	5.00	Thr (%)	0.73
_L_-Lys•HCl	0.31	Trp (%)	0.22
_L_-Thr	0.01	Available P (%)	0.36
_L_-Trp	0.17	Ca (%)	0.80
_DL_-Met	0.04		
Limestone	0.87		
Dicalcium phosphate	0.64		
Choline chloride	0.15		
NaCl	0.30		
Compound premix^a^	1.00		
Total	100.00		

^**a**^ The premix provides per kilogram of basal diet: copper 6.0 mg; iodine 0.14 mg; iron 120 mg; manganese 40 mg; selenium 0.3 mg; zinc 110 mg; vitamin A 2200 IU; vitamin K_3_ 0.5 mg; vitamin E 16 IU; vitamin D_3_ 220 IU; pantothenic acid; 10 mg; riboflavin 3.5 mg; niacin 30 mg; folic acid 0.3 mg. Thiamine 1 mg; pyridoxine 7 mg; biotin 0.05 mg; vitamin B_12_ 0.18 mg; antioxidant 150 mg; and anti-mold 50 mg

^b^ The crude protein and crude fiber in nutrient levels are measured values, and the rest are calculated values. Digestive energy of diets was calculated based on the databank of China Feed Ingredients (2020)

### Sample collection and measurement

BW and feed intake were monitored weekly, average daily gain, average daily feed intake and feed conversion ratio were calculated. The feces of piglets were observed and diarrhoea incidence were recorded daily according to Konieczka et al. [22]. The diarrhea rate (%) was calculated as the number of piglets with diarrhea / (number of pigs × number of test days) × 100. On the 28th of the experiment, Titanium dioxide (TiO_2_) was included in all diets in a 0.1% proportion to act as an indicator. After administering the diet with the indicator for 4 days, approximately 100 g of fecal samples were collected from each piglet for 3 consecutive days and stored at − 20 °C. The feed and fecal samples were measured for dry matter (DM), crude protein (CP), ether extract (EE) and crude fiber (CF) in accordance with AOAC (2017) methods. The ATTD of nutrients was calculated by equation below: ATTD (%) = 1 – {(A1 × F2)/ (A2 × F1)} × 100%, where A1 represents TiO_2_ content in feed while A2 denotes TiO_2_ content in feces, F1 indicates nutrient content in feed whereas F2 signifies nutrient content in feces.

On the 35th of the experiment, all piglets were weighed and euthanized by administering sodium pentobarbital intravenously (50 mg/kg·BW) for anesthesia after overnight fasting. The blood samples were collected into non-heparinized and EDTA vacuum tubes, centrifuged at 3000 g for 10 min at 4 °C, and the serum and plasma were frozen at -20 °C for subsequent ELISA and biochemical analysis. The jejunum and ileum were excised and promptly washed with phosphate-buffered saline (PBS) and chilled on ice. About 2 cm jejunal and ileal segments were isolated and then fixed in 4% formaldehyde for analysis of histomorphology. The jejunal and ileal mucosa were rinsed with cold saline, scraped gently with a slide and collected, rapidly frozen in liquid N_2_ and stored at -80 °C for gene expression and transcriptome analysis. About 5 g of colonic digesta was collected into sterile centrifuge tube, promptly frozen in liquid N_2_ and then stored at − 80 °C for the 16 S rDNA sequencing.

### Morphological analysis

Saline buffered 4% paraformaldehyde-fixed jejunum and ileum segments were embedded in paraffin, and then sectioned at 5 μm thickness were stained with H&E for micromorphological observation including crypt depth (CD) and villus height (VH) that were measured using cellSens Standard software V4.1.1 (Olympus Optical Company, Shenzhen, China).

### RNA isolation, qRT- PCR and sequencing analysis

Total RNA was extracted from mucosa of the small intestine (*n* = 6) by using Trizol^®^ reagent (Invitrogen™). The concentration and purity (OD260/280 = 1.8–2.2, OD260/230 ≥ 2.0) of total RNA were estimated using NanoDrop (P330, Munich, Germany). Evo M-MLV RT Kit with gDNA Clean (Accurate Biotechnology CO., LTD, ChangSha, China) was used to synthesize cDNA. Real-time PCR was utilized to measure the mRNA expression of nutrient transporters including *GLUT2*, *SGLT1*, *SLC1A1*, *SLC7A1*, *SLC7A7*, *SLC38A2*, *PEPT1*, *FABP4*, and *FATP-1*. The primer oligonucleotides listed in Table 2 were obtained from Sangon Biotech Co., Ltd. (Shanghai, China). The 2^−ΔΔCt^ method was employed to determine the expression of the target gene.

**Table 2:** Real-time PCR primers and amplified PCR product size for piglets

Gene	Primer sequence (5′- 3′)	Serial number	PCR Products size (bp)
*GLUT2*	F: ATTGTCACAGGCATTCTTGTTAGTCA	NM_001097417.1	273
	R: TTCACTTGATGCTTCTTCCCTTTC		
*SGLT1*	F: TCATCATCGTCCTGGTCGTCTC	NM_001164021.1	144
	R: CTTCTGGGGCTTCTTGAATGTC		
*SLC1A1*	F: GGCACCGCACTCTACGAAGCA	NM_001164649.1	177
	R: GCCCACGGCACTTAGCACGA		
*SLC7A1*	F: TCTGGTCCTGGGCTTCATAA	XM_021065165.1	123
	R: ACCTTCGTGGCATTGTTCAG		
*SLC7A7*	F: CTCGGGCATCTTCGTCT	XM_013978228.2	126
	R: CCCAGTTCCGCATAACA		
*SLC38A2*	F: GTTACCTTTGGTGATCCAGGC	XM_013997964.2	96
	R: ACCAATGACACCAGCAGAACC		
*PEPT1*	F: CAGACTTCGACCACAACGGA	NM_214347.1	99
	R: TTATCCCGCCAGTACCCAGA		
*FABP4*	F: TGGAAACTTGTCTCCAGTG	NM_001002817.1	147
	R: GGTACTTTCTGATCTAATGGTG		
*FATP-1*	F: GGAGTAGAGGGCAAAGCAGG	XM_021076151.1	208
	R: AGGTCTGGCGTGGGTCAAAG		
*ACTβ*	F: TGGAACGGTGAAGGTGACAGC	XM_003124280.5	177
	R: GCTTTTGGGAAGGCAGGGACT		

Abbreviations: *GLUT2* = solute carrier family 2 member 2; *SGLT1* = solute carrier family 5 member 10; *SLC1A1* = solute carrier family 1 member 1; *SLC7A1* = solute carrier family 7 member 1; *SLC38A2* = Solute carrier family 38 member 2; *PEPT1* = Solute carrier family 15 member 1; *FABP4* = fatty acid binding protein 4; *FATP-1* = fatty acid transport protein 1; *ACTβ* = actin beta

A total of 5 µg RNA per ileal sample (*n* = 5) was assessed for quality using a Bioanalyzer 2100 (Agilent Technologies, USA) and used for RNA sequencing library analysis with the RNA sample preparation Kit from Illumina (San Diego, CA, USA). The subsequent sequencing was conducted using the Illumina HiSeq 4000 (Majorbio, Shanghai, China). The processing procedures of raw sequencing data were displayed in the Appendix Information. The differentially expressed genes (DEGs) were qualified (*P* ≤ 0.05 and log_2_FC ≥ 1).

### Analysis of microbiota

The genomic DNA (gDNA) of colon content was extracted by the cetyltrimethylammonium bromide (CTAB). The primers F341 and R 806 (F: 5’-CCTAYGGGRBGCASCAG; R: 5’-GGACTACNNGGGTATCTAAT-3’) were used in the amplification of 16 S rRNA gene V3-V4 region in gut microbiota. The purity and concentration of the gDNA extracted were assessed using 2% agarose gel electrophoresis after amplification. Subsequently, the PCR products were purified and recovered for quantitative determination using the QIAquick PCR Purification kit (QIAGEN) and sequenced by Illumina NovaSeq 6000 platform (Novogene, Tianjin, China). After the run, the raw data were were filtered and noise reduced by FASTP and DADA2 to obtain amplicon sequence variants (ASVs). And then, each ASV were species annotated by the QIIME2 classify-sklearn algorithm using a pre-trained Naive Bayes classifier. Tukey’s test was calculated in the Alpha diversity (chao1 and Shannon index). Through Linear discriminant analysis Effect Size(LEfSe) and KEGG functional cluster abundance analyses, the changes in functions in different treatment groups were analyzed.

### Statistical analysis

All experimental results were analyzed by one-way ANOVA using the SPSS 29.0 statistical software package (SPSS). Duncan’s multiple comparisons test was utilized to assess the statistical differences among different treatments. The data are expressed as mean ± SEM. A value of *P* < 0.05 was considered statistically significant, while a value of 0.05 < *P* < 0.10 was considered a trend towards a difference. Correlations were analyzed by using spearman correlation analysis.

## Results

### Performance, diarrhea rate and apparent total tract digestibility

As shown in Table 3, the final BW of piglets tended to increase (*P* = 0.062) in 5-HTP treatments. Compared with CON group, piglets fed 250 mg/kg 5-HTP had higher (*P* < 0.05) ADG and lower (*P* < 0.05) FCR. There were no significant differences in average daily feed intake (ADFI) (*P* > 0.05) among the three groups. The 250 mg/kg group had lower (*P* < 0.05) diarrhea rate compared with the CON group. The ATTD of piglets is shown in Table 4. 5-HTP supplementation tended to decrease (*P* = 0.079) the apparent digestibility of EE. The CP digestibility in the 500 mg/kg group was lower (*P* < 0.05) compared with the 250 mg/kg group.

**Table 3:** Effect of dietary 5-HTP supplementation on growth performance of weaned piglets

Items	Treatments			SEM	*P* value
	CON	250 mg/kg	500 mg/kg		
Initial BW (kg)	8.30	8.30	8.25	0.243	0.996
Final BW (kg)	21.76	24.95	23.02	0.559	0.062
Average daily gain (g)	384.91^b^	475.67^a^	422.16^ab^	14.544	0.032
Average daily intake (g/ day)	777.23	817.85	834.43	14.160	0.231
Feed conversion ratio	2.05^a^	1.74^b^	1.98^ab^	0.052	0.034
Diarrhea rate (%)	22.03^a^	10.71^b^	16.67^ab^	1.504	0.002

^abc^ Means within a row without a common letter differ (*P* < 0.05), the same as below. Feed conversion ratio = average daily feed intake/average daily gain

**Table 4:** Effect of dietary 5-HTP supplementation on the apparent digestibility of weaned piglets

Items	Treatments			SEM	*P* value
	CON	250 mg/kg	500 mg/kg		
Dry matter (%)	78.46	79.13	75.70	0.826	0.206
Organic matter (%)	80.53	80.41	77.53	0.824	0.254
Crude protein (%)	68.32^ab^	70.45^a^	61.65^b^	1.397	0.016
Ether extract (%)	83.57	85.66	78.41	1.429	0.079
Crude fiber (%)	23.78	21.40	21.66	1.899	0.869

### Blood profile

As shown in Table 5, the group supplemented with 5-HTP resulted in an increase (*P* < 0.001) in serum 5-HT and IGF-1 levels. The serum GH concentration increased (*P* < 0.001) in 250 mg/kg group, nevertheless decreased (*P* < 0.001) in 500 mg/kg group. 5-HTP supplementation at a dose of 250 mg/kg notably enhanced the plasma TP and ALB concentrations, while 500 mg/kg did not exert a significant effect.

**Table 5:** Effect of dietary 5-HTP supplementation on blood hormones and biochemical parameters of weaned piglets

Items	Treatments				SEM	*P* value
	CON	250 mg/kg		500 mg/kg		
Serum						
5-HT (pg/mL)	370.45^b^	503.89^a^	572.56^a^		23.441	< 0.001
Growth hormone (ng/mL)	3.37^b^	4.52^a^	3.04^c^		0.157	< 0.001
Insulin-like growth factor-I (ng/mL)	75.20^c^	151.10^a^	117.74^b^		7.579	< 0.001
Plasma						
Total protein (g/L)	47.45^b^	53.33^a^	48.82^b^		0.867	0.006
Albumin (g/L)	29.53^b^	35.17^a^	31.6^b^		0.688	<0.001
Lactate dehydrogenase (U/L)	623.33	630.50	540.17		20.867	0.145
Glucose (mmol/L)	6.30	6.83	6.06		0.271	0.524
Calcium (mmol/L)	2.61	2.76	2.62		0.034	0.127

### Intestinal morphology

**Fig. 1:**
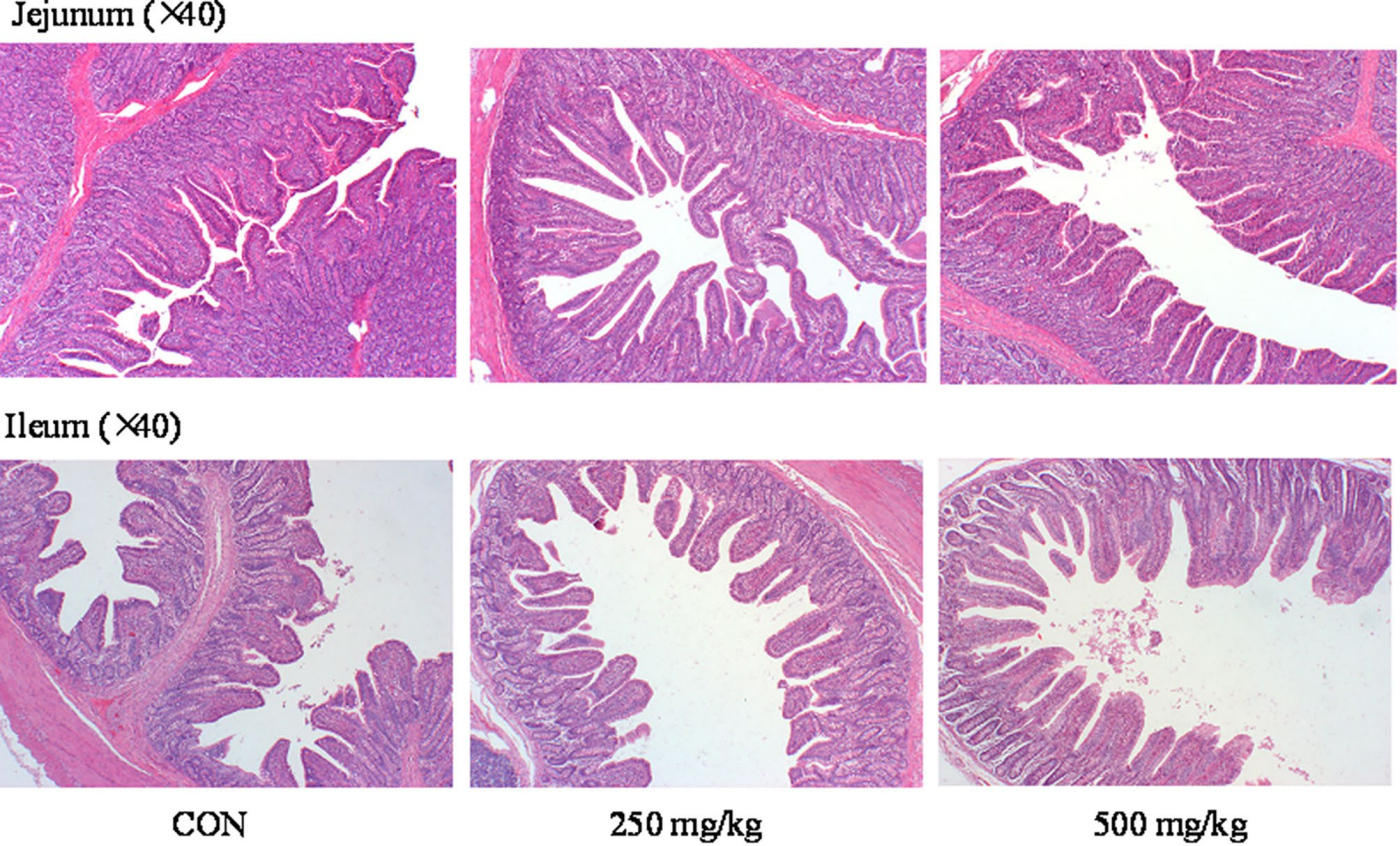
Effect of dietary 5-HTP supplementation on small intestinal morphology of piglets. Representative images showing HE staining microscopy results (40×)

As shown in Tables 5 and 6-HTP supplementation increased (*P* < 0.05) the jejunal VH compared to CON group. The addition of 5-HTP led to increase (*P* < 0.05) the ratio of V/C in jejunum. There were no significant differences in ileal VH and CD among the groups (*P* > 0.05) whereas ileal V/C ratio in the 250 mg/kg exhibited a greater value (*P* < 0.05) compared to the CON group. HE-stained sections revealed that the intestinal villus structure in 250 mg/kg group was more intact and denser than that of the CON group (Fig. 1).

**Table 6:** Effect of dietary 5-HTP supplementation on intestinal morphology of weaned piglets

Items	Treatments			SEM	
	CON	250 mg/kg	500 mg/kg		*P* value
Jejunum					
VH (µm)	278.76^b^	333.24^a^	313.65^a^	10.170	0.040
CD (µm)	133.81	117.14	137.88	4.307	0.110
V/C	2.13^c^	2.94^a^	2.46^b^	0.095	<0.001
Ileum					
VH (µm)	271.20	319.64	309.15	11.295	0.188
CD (µm)	144.50	123.90	124.65	6.500	0.362
V/C	1.93^b^	2.67^a^	2.51^a^	0.130	0.039

Abbreviations: VH: villus height; CD: crypt depth; V/C: villus height/crypt depth

### Ileal transcriptomic analysis

**Fig. 2:**
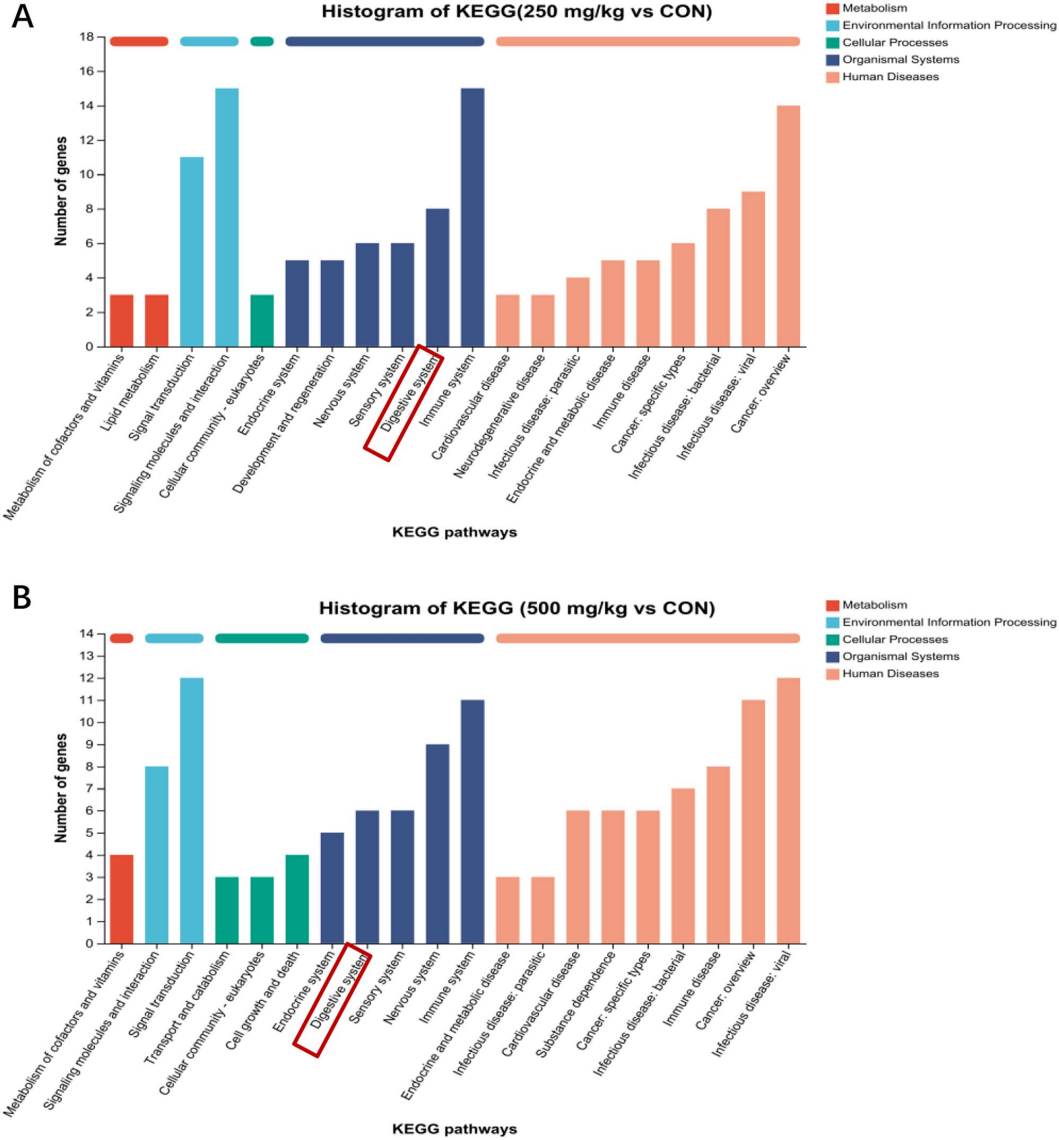
The modulation of 5-HTP on ileal transcriptomics. (**A**) The kyoto encyclopedia of genes and genomes (KEGG) classification of DEGs between CON and 250 mg/kg. (**B**) The KEGG classification of DEGs between CON and 500 mg/kg

**Fig. 3:**
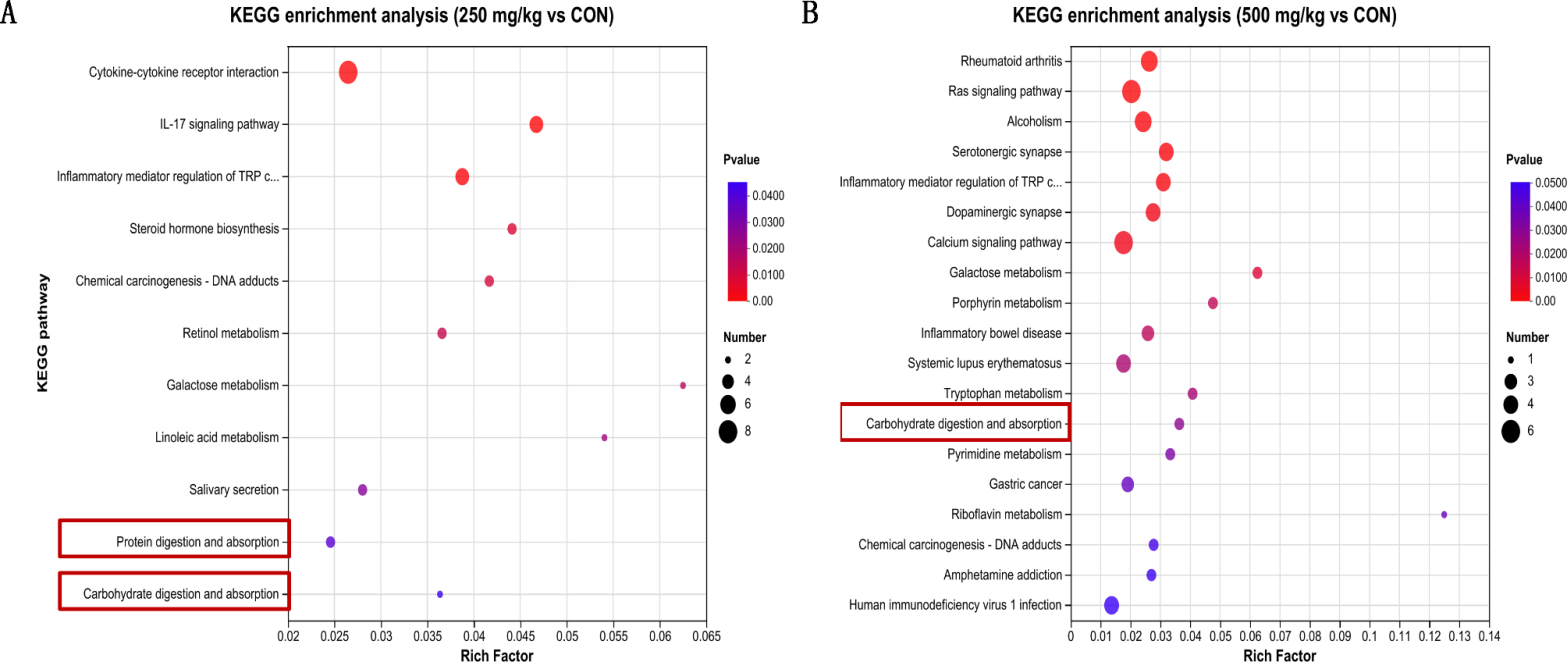
The modulation of 5-HTP on ileal transcriptomics. (**A**) The KEGG pathway enrichment of DEGs between CON and 250 mg/kg. (**B**) The pathway enrichment of DEGs between CON and 500 mg/kg

**Fig. 4:**
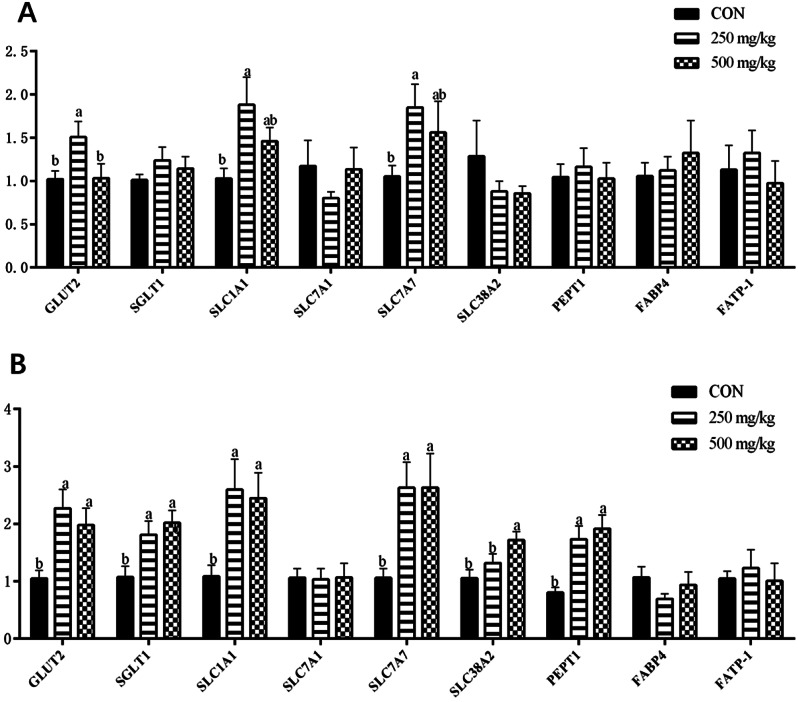
The modulation of 5-HTP on mRNA expression of nutrient transport-related genes in intestine. (**A**) Effect of 5-HTP on mRNA expression of nutrient transport-related genes in jejunal mucosa. (**B**) Effect of 5-HTP on mRNA expression of nutrient transport-related genes in the ileal mucosa

The specific DEGs between CON_250 mg/kg and CON_500 mg/kg comparisons were filtered out by using Venn diagram analysis. Overall 71 DEGs specifically respond to the CON group, 211 DEGs specifically respond to the 250 mg/kg group, 180 DEGs specifically respond to the 500 mg/kg group (Appx. Figure 1 A). A heatmap of the DEGs revealed significant alterations in gene expression patterns among the groups (Appx. Figure 1B). The KEGG enrichment analysis was performed with those DEGs between two groups. Results showed that DEGs were primarily categorized into four domains: organismal systems, metabolism, cellular processes and environmental information at the first level (Fig. 2A-B). Interestingly, the shared predominant KEGG subclass in organismal systems were immune system and digestive system in “250 mg/kg vs. CON” and “500 mg/kg vs. CON”. Protein digestion and absorption and Carbohydrate digestion and absorption both belonged to the digestive system (Fig. 3A-B). To further determine whether 5-HTP could regulate protein and carbohydrate digestion and absorption in piglets, we verified several genes related to nutrient transport by quantitative real-time PCR (Fig. 4A-B). The 250 mg/kg supplementation of 5-HTP up-regulated *GLUT2*, *SLC7A7*, and *SLC1A1* expression, while 500 mg/kg supplementation of 5-HTP up-regulated *SLC7A7* and *SLC1A1* expression in jejunal mucosa (*P*<0.05). Dietary 5-HTP supplementation up-regulated (*P*<0.05) *GLUT2*, *SGLT1*, *SLC1A1*, *SLC7A7*, and *PEPT1* expression in ileal mucosa. Also, the expression of *SLC38A2* was up-regulated (*P* < 0.05) in 500 mg/kg group in ileal mucosa.

### Gut microbiota

**Fig. 5:**
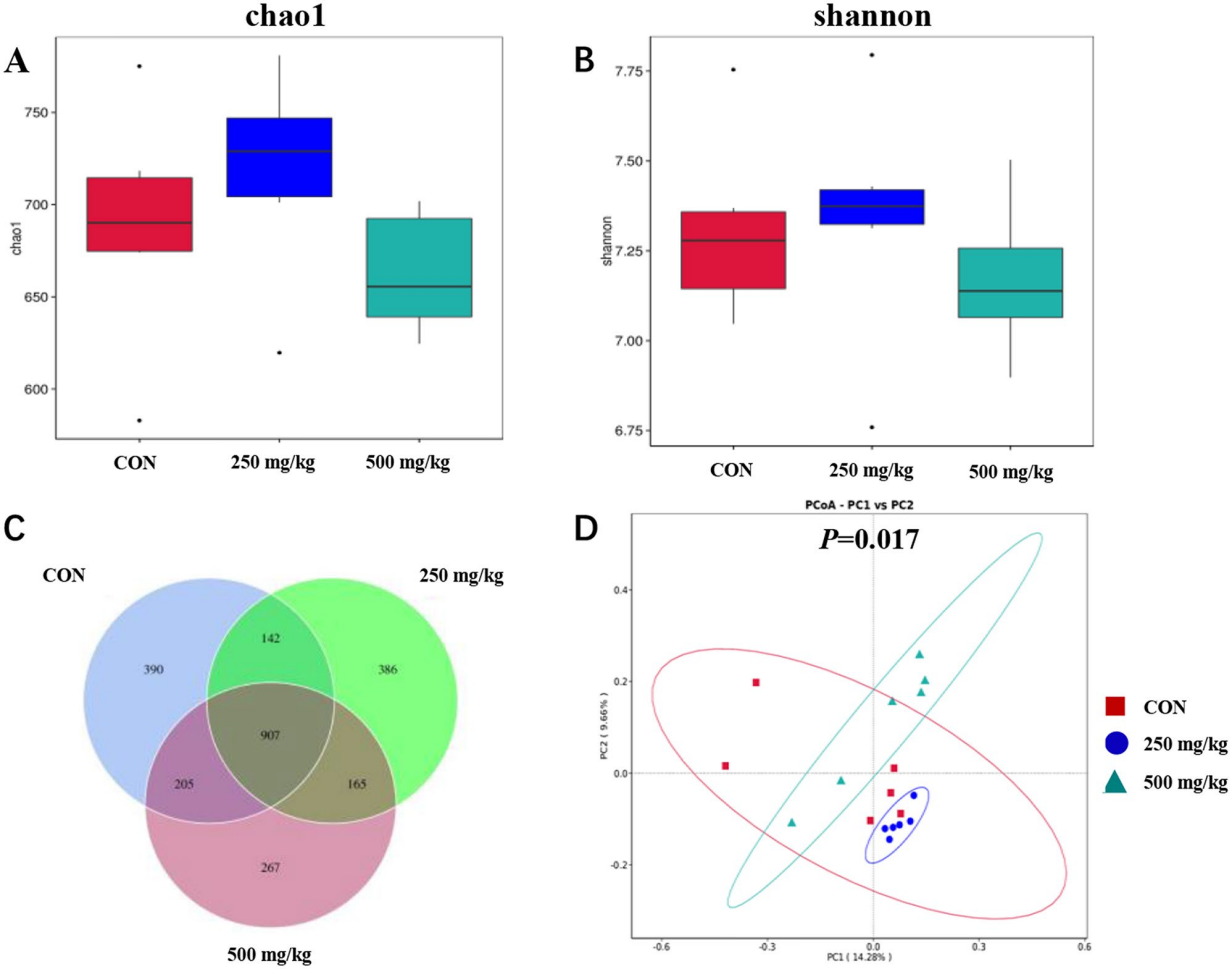
The modulation of 5-HTP on colon microbial community. (**A**) Chao1 index (**B**) Shannon index (**C**) The venn diagram between CON, 250 and 500 mg/kg groups. (**D**) Principal component analysis (PCA) in the CON, 250 and 500 mg/kg groups according to the Bray Curtis distance metric. Abbreviations: **A**: CON; **B**: 250 mg/kg; **C**: 500 mg/kg

**Fig. 6:**
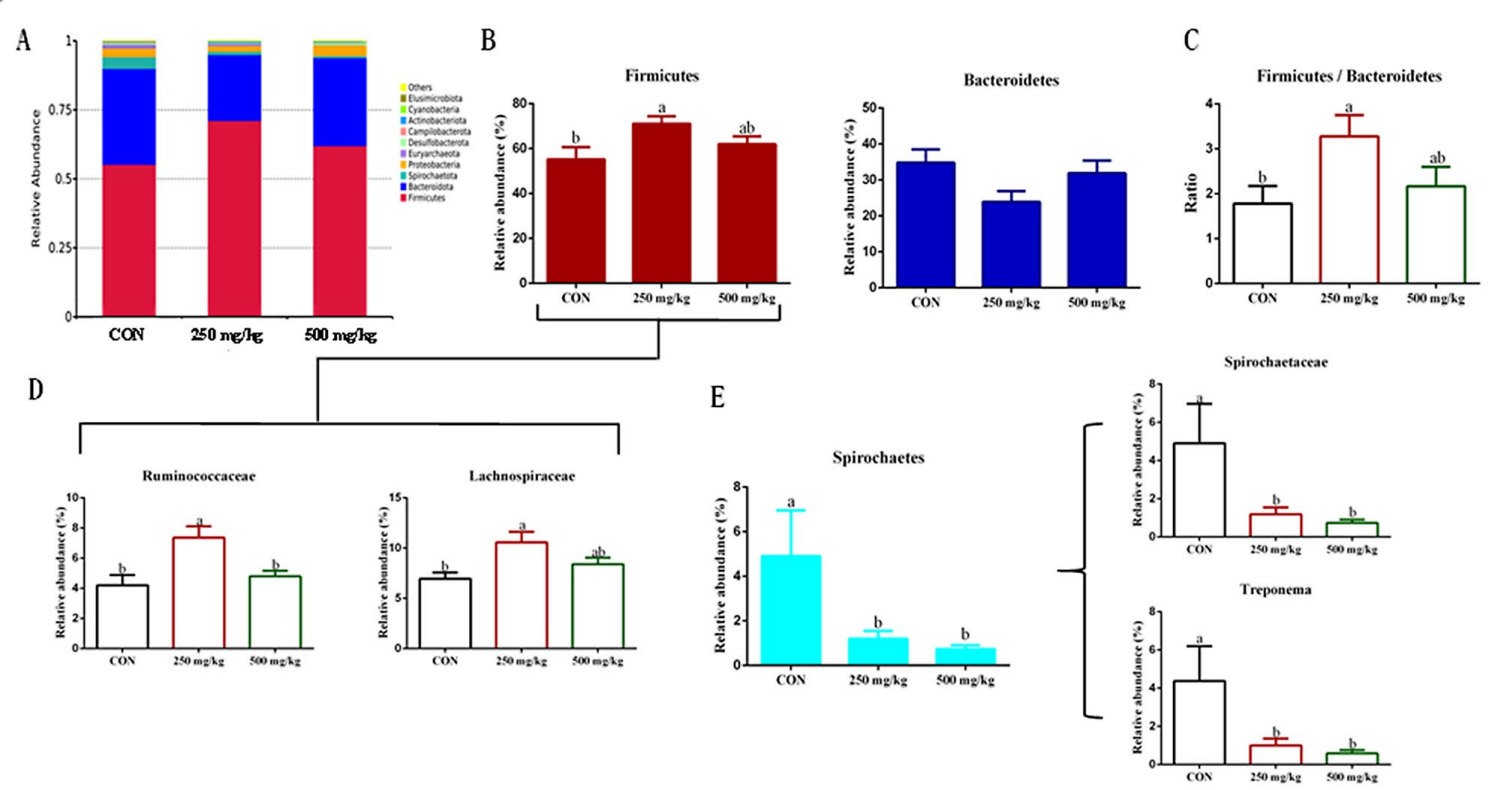
The modulation of 5-HTP on colon bacterial communities in piglets. (**A**) The phylum-level taxonomic composition of gut bacterial communities in piglets of different group (abbreviations: **A**: CON; **B**: 250 mg/kg; **C**: 500 mg/kg). (**B**) Effects of dietary 5-HTP supplementation on the phylum level abundance of *Firmicutes* and *Bacteroidetes*. (**C**) The Firmicutes/Bacteroidetes ratio in each group. (**D**) Effects of dietary 5-HTP supplementation on the phylum level abundance of *Lachnospiracea* and *Ruminococcaceae* which belong to *Firmicutes*. (**E**) Effects of dietary 5-HTP supplementation on the relative abundance of *Spirochaetes*, as well as family and genera including

**Fig. 7:**
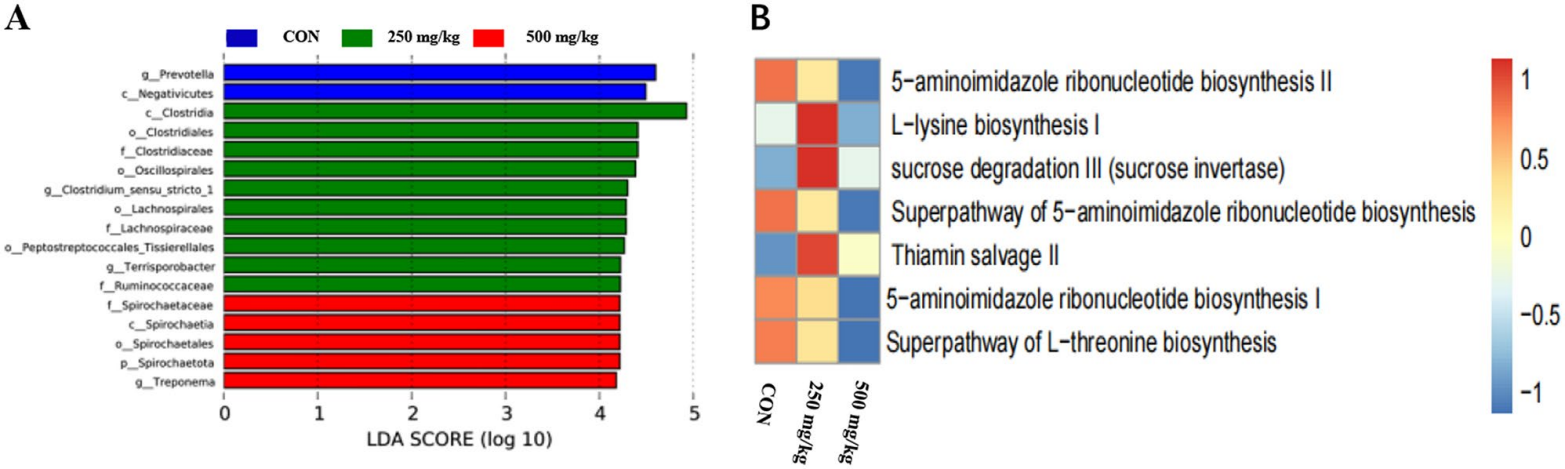
The modulation of 5-HTP on colon bacterial communities in piglets. (**A**) LEfSe analyses. (**B**) The KEGG pathway analysis of the predicted functions of the colonic microbiota. Abbreviations: **A**: CON; **B**: 250 mg/kg; **C**: 500 mg/kg

**Fig. 8:**
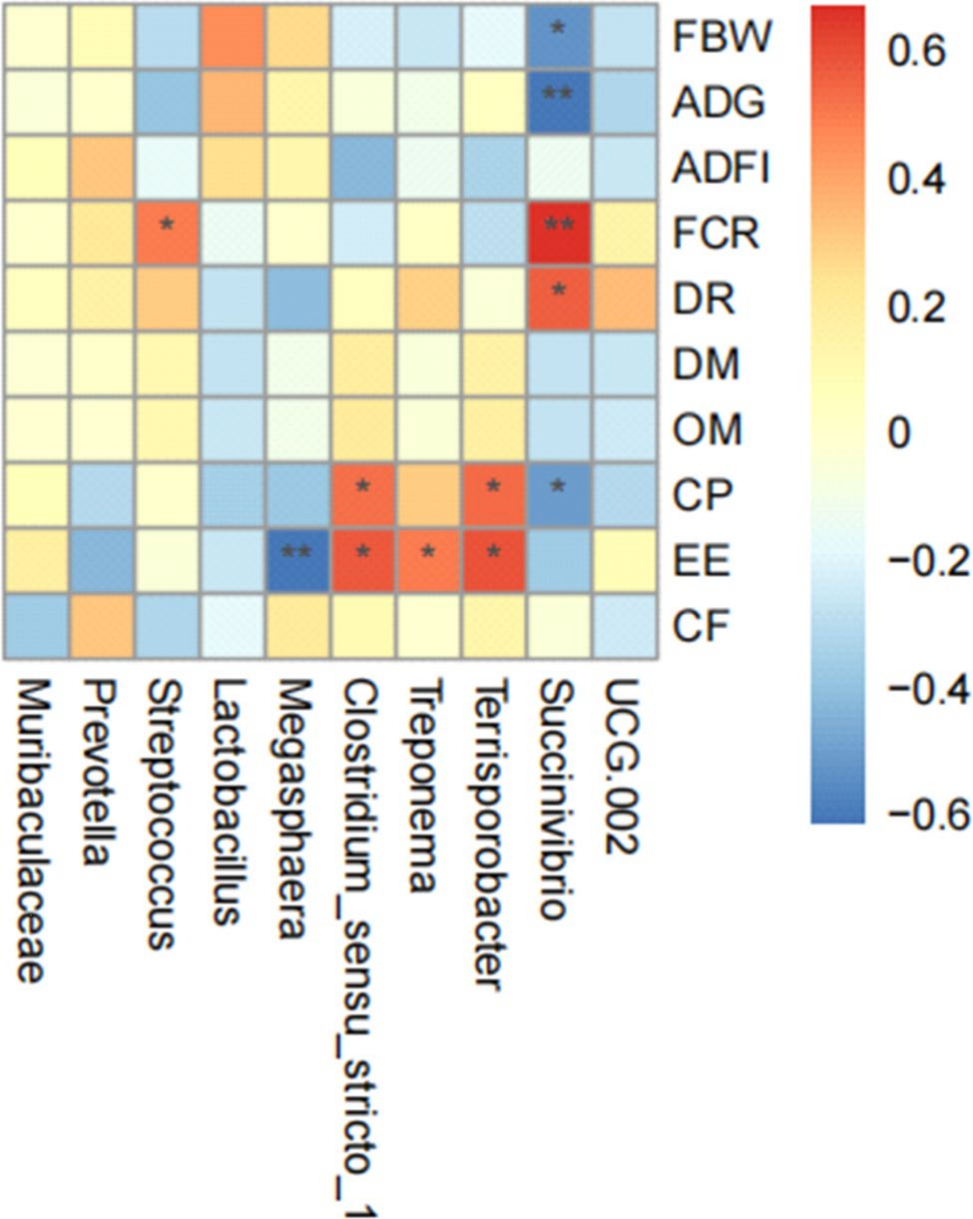
Correlation analysis between growth performance and altered microbiota. Heatmap of Spearman’s correlation between growth performance, ATTD and gut microbiota. *, *P* < 0.05; **, *P* < 0.01. Abbreviation: DR = diarrhea rate

The effects of dietary 5-HTP supplementation on gut microbiota in weaned piglets are presented in Fig. 5. The alpha diversity analysis revealed no significant differences in the Chao1 and Shannon indexes among the treatment groups. There were 907 core ASVs observed in the Venn diagram of the three groups. 390, 386, 267 elements were unique to the CON, 250 and 500 mg/kg groups, respectively. Nevertheless, the principal component analysis (PCA) (Fig. 5D) demonstrated a notable impact of 5-HTP on gut microbiota composition, with a distinct difference observed among the clusters of the treatment groups (*P* = 0.017). Supplementation with 250 mg/kg 5-HTP had a higher (*P* < 0.05) relative abundance of *Firmicutes*, leading to an increased (*P* < 0.05) *Firmicutes*/*Bacteroidetes* ratio (Fig. 6C). In addition, the abundance level of *Actinobacteriota* increased (*P* < 0.05) in 500 mg/kg group (Appx. Table 1). Further analysis revealed that *Lachnospiracea* and *Ruminococcaceae* at the family level were identified as marker species in 250 mg/kg group by LEfSe analysis (Figs. 6D and 7A). The 5-HTP supplementation groups decreased the abundance of *Spirochaetes* at the phylum level, while *Spirochaetaceae* and *Treponema* were significantly down regulated by 5-HTP at family and genus level (*P* < 0.05). Meanwhile, the CON group was enriched with *Spirochaetaceae* (family) and *Treponema* (genus) in LEfSe analysis (Fig. 7A). Additionally, the abundance level of *Megasphaera* was increased (*P* < 0.05) in 500 mg/kg group (Appx. Table 2). The KEGG analysis revealed that sucrose degradation, L-lysine biosynthesis and Thiamin salvage were enriched in 250 mg/kg group compared with CON group in PICRUSt analysis, while metabolism of L-lysine synthesis and 5-aminoimidazole ribonucleic acid synthesis were enriched in the CON compared with 500 mg/kg group (Fig. 7B). To further understand the role of gut microbiota in improving growth performance, the genera with relative abundance in top 10 were selected for correlation with growth performance and ATTD indicators (Fig. 8). The genus *Streptococcus* was positively correlated with FCR (*P* < 0.05). The genus *Megasphaera* was negatively correlated with the ATTD of EE (*P* < 0.01). Both genus *Clostridium_sensu_stricto_1* and *Terrisporobacter* were positively correlated with the ATTD of CP and EE (*P* < 0.05).The genus *Treponema* was positively correlated with the ATTD of EE (*P* < 0.05). The genus *Succinivibrio* was negatively correlated with final BW, ATTD of CP(*P* < 0.05) and ADG (*P* < 0.01). The genus *Succinivibrio* was positively correlated with diarrhea rate (*P* < 0.05) and FCR (*P* < 0.01).

## Discussion

After early weaning, piglets commonly suffer from weaning stress, which has a considerable influence on long-term intestinal health and growth performance during finishing period [2]. In this study, supplementation with 5-HTP, which is a direct precursor of 5-HT, resulted in an increased final BW and ADG, while reducing the FCR in piglets. This might be attributed to the role of 5-HT as a neurotransmitter, which modulates emotions and motivation, thereby alleviating weaning stress in piglets [23]. In addition, the growth-promoting function of 5-HT is related to the GH axis. Previous studies have shown that administration of 5-HT into rats’ hypothalamus can increase their serum GH levels [24]. Other research has shown that GH secretion is stimulated by peripheral administration of quipazine, an agonist of 5-HT receptors in cattle [25]. Furthermore, dietary supplementation of Trp can enhance basal GH secretion in weaned piglets [6]. In the present study, supplementing piglet diets with 250 mg/kg 5-HTP increased the levels of 5-HT, GH and IGF-1 in serum. This result further suggests that 5-HTP supplementation can be effectively converted to 5-HT in vivo and improve the piglets’ growth performance through the GH-IGF-1 axis.

The V/C ratio has been widely recognized as a measured indicator for assessing the morphological integrity and functional capacity of digestion and absorption in the small intestine [26]. Intestinal VH is positively correlated with nutrient absorption capacity [27]. In this study, piglets fed diets supplemented with 5-HTP increased VH both in jejunum and ileum. Similarly, feeding 5-HTP diets resulted in higher V/C ratio in jejunum and ileum, which is an important factor in promoting postweaning growth. These results parallel the influence of the dietary Trp on small intestinal morphology, which showed that dietary Trp (0.2–0.5%) supplementation increased villus height and decreased crypt depth, as a result, the ratio of V/C increased [28, 29]. However, supplementation of high concentrations of Trp can impair the intestinal development of weaned piglets. Dietary 0.75% Trp increased the crypt depth of jejunum, accompanied by a significant decrease in the V/C ratio in piglets [29]. In the present study, we observed that the VH and V/C ratio at 500 mg/kg level were lower than those in 250 mg/kg level. This indicates that excessive supplementation of dietary Trp may undermine the integrity of the gut barrier in piglets. 5-HTP is the intermediate metabolite of the essential amino acid L-Trp in the biosynthesis of 5-HT, indicating that the negative effects on intestinal morphology caused by supplementation with either excessive Trp or 5-HTP may be induced by their final downstream product, 5-HT. Thus, the dietary of 250 mg/kg 5-HTP improved intestinal morphology and resulted in better growth performance and feed efficiency in weaned piglets.

It has been well established that gut-derived 5-HT plays an important role in the regulation of GIM by mediating excitatory neurotransmission [30]. Gastrointestinal motility (GIM) is a key factor that influences the proliferation of the intestinal microbiome [31]. The mucus secretions, along with GIM, act as a defense mechanism of the epithelium by propelling pathogens and toxins along the gut lumen for eventual elimination. However, an increase in GIM within the small intestine could potentially lead to higher intestinal permeability [32]. The release of 5-HT from damaged intestinal mucosa in allergic reaction enhances the strength and speed of GIM, thereby contributing to the development of diarrhea [33]. The frequency of defecation increased after injecting with 5-HT in cows [34]. There was no further improvement in diarrhea in the 500 mg/kg group in the present study. Meanwhile, dietary 5-HTP supplementation at dose of 500 mg/kg significantly decreased the ATTD of CP and EE. These results suggested that piglets fed 500 mg/kg 5-HTP may reduce the retention time of the chyme in gastrointestinal tract and decrease the ATTD of nutrients. To further explore the influence of 5-HTP on intestinal absorption function, we analyzed the ileal transcriptome and detected the mRNA expression of nutrient transporters in jejunal and ileal mucosa. Interestingly, we found that dietary 5-HTP supplementation improved intestinal absorption function by up-regulated the expression of nutrient transporters. This result was in line with the beneficial effects of 5-HTP on intestinal morphology and TP and ALB level in plasma, which suggesting that 5-HTP improved intestinal absorption and body protein biosynthesis. Taken together, these findings provide evidence that supplementing 5-HTP at 250 mg/kg level enhances the capacity of nutrients utilization, resulting in improved growth performance. However, the increased gene expression of nutrient transporters in 500 mg/kg group may be a compensatory response caused by decreased nutrient digestibility, and the mechanism needs to be investigated in future studies.

The weaning stage of piglets is an important window period to shape the gut microbiota [35]. The intestinal microflora balance of piglets is maintained by the metabolic activity of gut microbiome and its metabolites [36]. In this study, the Venn diagram analysis demonstrated a substantial overlap in the microbiome among all samples from the three groups. However, each group also exhibited its distinct microbial composition.The *Firmicutes* were more dominant in nutrient absorption and subsequent weight gain than *Bacteroidetes* [37, 38]. Our findings revealed that 5-HTP supplementation resulted in an increased *Firmicutes*/*Bacteroidetes* ratio, which was consistent with the growth performance of piglets. Furthermore, the observed increase in *Firmicutes* was attributed to the greater abundance of *Megasphaera*, which possesses ability to promote intestinal carbohydrate metabolism balance and effectively prevent diarrhea associated with hyper-lactate accumulation in piglets [39]. This was consistent with our study that the 5-HTP decreased diarrhea rate in the 250 mg/kg group. However, the diarrhea rate remained unchanged despite the increased abundance of *Megasphaera* in 500 mg/kg group. Due to the potential neurotransmitter properties of 5-HT in promoting GIM, the flourishing of this microorganism in the 500 mg/kg group, which improves the host’s ability to prevent diarrhea, may be offset by 5-HT-induced GIM, and the underlying mechanism needs further study. Based on correlation analysis at the genus level, *Clostridium_sensu_stricto_1* and *Terrisporobacter* were positively correlated with the ATTD of CP and EE. On the one hand, *Clostridium_sensu_stricto_1* has been reported to produce butyrate and promote the adhesion of the intestinal mucosal barrier to pathogens [40]. On the other hand, *Terrisporobacter* maintain gut homeostasis with its antimicrobial properties in cecal microbial community [41]. These findings may reveal that both *Clostridium_sensu_stricto_1* and *Terrisporobacter *stabilize the intestinal environment with its fermentation products to improve intestinal digestion during piglet weaning stress. In addition, the family of *Ruminococcaceae* and *Lachnospiraceae* has been inhabited by 5-HTP, which exert probiotic physiological functions by producing short-chain fatty acids in the gut [42, 43]. It has also been reported that *Succinivibrio* which was positively correlated with the FCR play a key role in feed efficiency by utilizing carbohydrates to break down short-chain fatty acids in the fermentation process of microorganisms [44]. Besides, we observed that 5-HTP significantly increased the abundance of *Actinobacteria* at the phylum level. *Actinobacteria* play an important role in maintaining gut homeostasis as well as potential therapeutic use for gastrointestinal pathological conditions and systemic diseases [45]. It has been observed that the abundance of *Actinobacteria* was reduced in patients with coeliac disease [46, 47]. Moreover, the 5-HTP addition group had a diminished abundance of *Spirochaetes* and *Treponema*, as well as the specific species in the CON group by LEfSe analysis. *Treponema*, as the sole genus within the phylum *Spirochaetes*, is considered a harmful microorganism that is pathogenic to pigs [48]. It causes dysentery and colitis by invading the surface epithelium and mucosa of the intestines [49]. A previous study indicated that dietary supplementation with 2% rapeseed protein led to lower diarrhea incidence and reduced the relative abundance *Treponema* in piglets [50]. Dietary lycopene could remarkably improve anti-inflammatory responses in piglets, which was related to a reduction in the abundance of *Treponema* [51]. Overall, these findings suggest that dietary 5-HTP supplementation makes an improvement in the growth performance of piglets attributed to probiotics with highly abundant colonic bacteria, i.e., *Firmicutes*, *Actinobacteria*, *Ruminococcaceae*, *Lachnospiraceae*, and *Megasphaera*. Additionally, 5-HTP suppressed potential pathogens related to diarrhea, such as *Spirochaetes* and *Treponema*, thereby promoting intestinal microbial balance.

## Conclusion

In conclusion, our study demonstrated that supplementation of 5-HTP at 250 mg/kg improved growth performance, reduced diarrhea rate, enhanced nutrient digestibility, associated with favorable alterations in intestinal microbiota of weaned piglets. These findings suggest the potential of using 5-HTP as a dietary supplement to enhance the intestinal health and productivity of weaned piglets.

## Figures and Tables

**Appendix Fig. 1:**
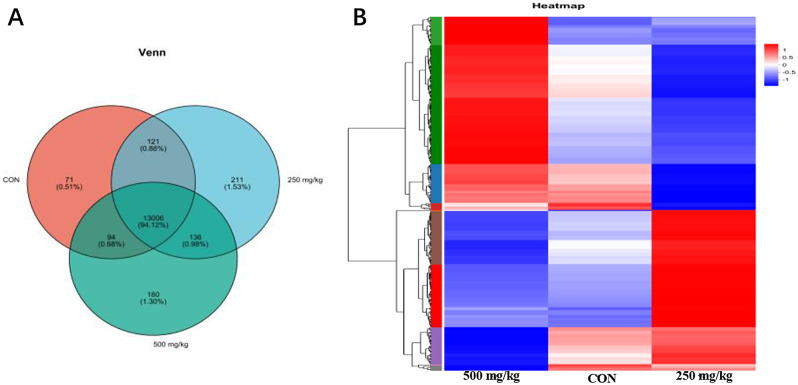
The modulation of 5-HTP on ileal transcriptomics. (**A**) The Venn diagram of DEGs (**B**) The heatmap of top DEGs between CON and 5-HTP addition groups

## Data Availability

No datasets were generated or analysed during the current study.

## Appendix

### Data processing and DEG screening

The raw sequencing data were initialy processed for quality control using FastQC and Trimmomatic by removing reads containing adapter, reads containing ploy-N, and low-quality reads, Q20, Q30, and GC-content were calculated for the clean reads. These clean reads were mapped to the pig reference genome (Sscrofa11.1) using HISAT2, and transcript assembly was performed with StringTie v 2.2.3. DEGs were identified using DESeq2 which provides statistical routines for determining differential expression in digital gene expression data using a model based on the negative binomial distribution. The resulting *P* values were adjusted using the Benjamini and Hochberg’s approach for controlling the false discovery rate. Genes with an adjusted *P*-value < 0.05 found using DESeq were assigned as differentially expressed. Functional annotation, including Gene Ontology (GO) enrichment and KEGG pathway analysis, was conducted to explore the biological processes and pathways affected by 5-HTP.

**Appendix Table 1:** Effects of dietary 5-HTP supplementation on the composition of colonic microbiota at the phylum levels (%)

Items	Treatments			SEM	
	CON	250 mg/kg	500 mg/kg		*P* value
*Firmicutes*	55.21^b^	71.11^a^	61.99^ab^	2.764	0.032
*Bacteroidetes*	34.79	23.88	31.91	2.181	0.101
*Spirochaetes*	4.90	1.19	0.74	0.728	0.034
*Proteobacteria*	3.14	2.11	3.89	0.502	0.369
*Euryarchaeota*	1.35	0.72	0.09	0.322	0.296
*Desulfobacterota*	0.59	0.34	0.79	0.088	0.105
*Campilobacterota*	0.34	0.06	0.15	0.059	0.137
*Actinobacteriota*	0.27^b^	0.49^a^	0.35^ab^	0.038	0.045
*Cyanobacteria*	0.01	0.02	0.04	0.008	0.372

**Appendix Table 2:** Effect of dietary 5-HTP supplementation on the composition of colonic microbiota at the genus levels (%)

Items	Treatments			SEM	
	CON	250 mg/kg	500 mg/kg		*P* value
*Prevotella*	8.60	5.36	13.59	1.557	0.087
*Muribaculaceae*	12.62	9.55	7.59	1.312	0.304
*Streptococcus*	8.35	7.68	10.04	0.940	0.601
*Lactobacillus*	5.03	6.42	8.56	0.861	0.254
*Megasphaera*	2.25^b^	3.98^ab^	7.10^a^	0.829	0.041
*Clostridium_sensu_stricto_1*	4.38	5.51	2.08	0.672	0.100
*Treponema*	2.25^a^	0.71^b^	0.58^b^	0.269	0.009
*Alloprevotella*	1.30	1.52	1.72	0.209	0.778
*Megamonas*	0.01	0.66	0.47	0.152	0.208
*Succinivibrio*	2.41	0.89	3.32	0.505	0.139

## References

[1] Lucas ME, Hemsworth LM, Hemsworth PH, Review. Early life piglet experiences and impacts on immediate and longer-term adaptability. Animal. 2023;18:100889. 37468352 10.1016/j.animal.2023.100889

[2] Lallès JP, Bosi P, Smidt H, Stokes CR. Nutritional management of gut health in pigs around weaning. Proc Nutr Soc. 2007;66(2):260–8. 17466106 10.1017/S0029665107005484

[3] HöTzel MJ, Souza G, Costa O, Filho L. Disentangling the effects of weaning stressors on piglets’ behaviour and feed intake: Changing the housing and social environment. Appl Anim Behav Sci. 2011;135(1–2):44–50.

[4] Keithahn C, Lerchl A. 5-hydroxytryptophan is a more potent in vitro hydroxyl radical scavenger than melatonin or vitamin C. J Pineal Res. 2005;38(1):62–6. 15617538 10.1111/j.1600-079X.2004.00177.x

[5] Choi W, Moon JH, Kim H. Serotonergic regulation of energy metabolism in peripheral tissues. J Endocrinol. 2020;245(1):R1–10. 32092036 10.1530/JOE-19-0546

[6] Rao Z, Li J, Shi B, Zeng Y, Liu Y, Sun Z, Wu L, Sun W, Tang Z. Dietary Tryptophan Levels Impact Growth Performance and Intestinal Microbial Ecology in Weaned Piglets via Tryptophan Metabolites and Intestinal Antimicrobial Peptides. Anim (Basel). 2021;11(3):817. 10.3390/ani11030817 PMC7999158 33799457

[7] Liu G, Lu J, Sun W, Jia G, Zhao H, Chen X, Kim IH, Zhang R, Wang J. Tryptophan Supplementation Enhances Intestinal Health by Improving Gut Barrier Function, Alleviating Inflammation, and Modulating Intestinal Microbiome in Lipopolysaccharide-Challenged Piglets. Front Microbiol. 2022;13:919431. 35859741 10.3389/fmicb.2022.919431 PMC9289565

[8] Liu G, Sun W, Wang F, Jia G, Zhao H, Chen X, Tian G, Cai J, Wang J. Dietary tryptophan supplementation enhances mitochondrial function and reduces pyroptosis in the spleen and thymus of piglets after lipopolysaccharide challenge. Animal. 2023;17(3):100714. 36764015 10.1016/j.animal.2023.100714

[9] Henry Y, Sève B, Mounier A, Ganier P. Growth performance and brain neurotransmitters in pigs as affected by tryptophan, protein, and sex. J Anim Sci. 1996;74(11):2700–10. 8923184 10.2527/1996.74112700x

[10] Zendehdel M, Sardari F, Hassanpour S, Rahnema M, Adeli A, Ghashghayi E. Serotonin-induced hypophagia is mediated via α(2) and β(2) adrenergic receptors in neonatal layer-type chickens. Br Poult Sci. 2017;58(3):298–304. 28362179 10.1080/00071668.2017.1278626

[11] Sargent BJ, Henderson AJ. Targeting 5-HT receptors for the treatment of obesity. Curr Opin Pharmacol. 2011;11(1):52–8. 21330209 10.1016/j.coph.2011.01.005

[12] Liang Q, Zhu B, Liu D, Lu Y, Zhang H, Wang F. Serotonin and dopamine regulate the aggressiveness of swimming crabs (Portunus trituberculatus) in different ways. Physiol Behav. 2023;263:114135. 36813219 10.1016/j.physbeh.2023.114135

[13] Oleskin AV, Shenderov BA, Rogovsky VS. Role of Neurochemicals in the Interaction between the Microbiota and the Immune and the Nervous System of the Host Organism. Probiotics Antimicrob Proteins. 2017;9(3):215–34. 28229287 10.1007/s12602-017-9262-1

[14] Reyes-Gonzales MC, Fuentes-Broto L, Martínez-Ballarín E, Miana-Mena FJ, Berzosa C, García-Gil FA, Aranda M, García JJ. Effects of tryptophan and 5-hydroxytryptophan on the hepatic cell membrane rigidity due to oxidative stress. J Membr Biol. 2009;231(2–3):93–9. 19847470 10.1007/s00232-009-9208-y

[15] Bae SJ, Lee JS, Kim JM, Lee EK, Han YK, Kim HJ, Choi J, Ha YM, No JK, Kim YH, et al. 5-Hydroxytrytophan inhibits tert-butylhydroperoxide (t-BHP)-induced oxidative damage via the suppression of reactive species (RS) and nuclear factor-kappaB (NF-kappaB) activation on human fibroblast. J Agric Food Chem. 2010;58(10):6387–94. 20415419 10.1021/jf904201h

[16] Chae HS, Kang OH, Choi JG, Oh YC, Lee YS, Jang HJ, Kim JH, Park H, Jung KY, Sohn DH, Kwon DY. 5-hydroxytryptophan acts on the mitogen-activated protein kinase extracellular-signal regulated protein kinase pathway to modulate cyclooxygenase-2 and inducible nitric oxide synthase expression in RAW 264.7 cells. Biol Pharm Bull. 2009;32(4):553–7. 19336883 10.1248/bpb.32.553

[17] Birdsall TC. 5-Hydroxytryptophan: a clinically-effective serotonin precursor. Altern Med Rev. 1998;3(4):271–80. 9727088

[18] Wang H, Liu S, Li J, Wang L, Wang X, Zhao J, Jiao H, Lin H. 5-Hydroxytryptophan Suppresses the Abdominal Fat Deposit and Is Beneficial to the Intestinal Immune Function in Broilers. Front Physiol. 2020;11:655. 32595527 10.3389/fphys.2020.00655 PMC7304481

[19] Wu L, Ran L, Wu Y, Liang M, Zeng J, Ke F, Wang F, Yang J, Lao X, Liu L, et al. Oral Administration of 5-Hydroxytryptophan Restores Gut Microbiota Dysbiosis in a Mouse Model of Depression. Front Microbiol. 2022;13:864571. 35572711 10.3389/fmicb.2022.864571 PMC9096562

[20] Jacobsen JPR, Oh A, Bangle R, Roberts WL, Royer EL, Modesto N, Windermere SA, Yi Z, Vernon R, Cajina M, et al. Slow-release delivery enhances the pharmacological properties of oral 5-hydroxytryptophan: mouse proof-of-concept. Neuropsychopharmacology. 2019;44(12):2082–90. 31035282 10.1038/s41386-019-0400-1 PMC6898594

[21] Telli G, Kazkayasi I, Uma S. The effects of 5-hydroxytryptophan on carrageenan-induced mouse paw oedemas. Rev Nutr. 2021;34:e200119.

[22] Konieczka P, Ferenc K, Jørgensen JN, Hansen LHB, Zabielski R, Olszewski J, Gajewski Z, Mazur-Kuśnirek M, Szkopek D, Szyryńska N, Lipiński K. Feeding Bacillus-based probiotics to gestating and lactating sows is an efficient method for improving immunity, gut functional status and biofilm formation by probiotic bacteria in piglets at weaning. Anim Nutr. 2023;13:361–72. 37388456 10.1016/j.aninu.2023.03.003 PMC10300407

[23] Gorka SM, Young CB, Klumpp H, Kennedy AE, Francis J, Ajilore O, Langenecker SA, Shankman SA, Craske MG, Stein MB, Phan KL. Emotion-based brain mechanisms and predictors for SSRI and CBT treatment of anxiety and depression: a randomized trial. Neuropsychopharmacology. 2019;44(9):1639–48. 31060042 10.1038/s41386-019-0407-7 PMC6785075

[24] Willoughby JO, Menadue MF, Liebelt H. Activation of serotonin receptors in the medial basal hypothalamus stimulates growth hormone secretion in the unanesthetized rat. Brain Res. 1987;404(1–2):319–22. 3567575 10.1016/0006-8993(87)91386-2

[25] Gaynor PJ, Lookingland KJ, Tucker HA. 5-Hydroxytryptaminergic receptor-stimulated growth hormone secretion occurs independently of changes in peripheral somatostatin concentration. Proc Soc Exp Biol Med. 1995;209(1):79–85. 7724619 10.3181/00379727-209-43881

[26] Tang X, Xiong K. Intrauterine Growth Retardation Affects Intestinal Health of Suckling Piglets via Altering Intestinal Antioxidant Capacity, Glucose Uptake, Tight Junction, and Immune Responses. Oxid Med Cell Longev. 2022, 2022:2644205. 10.1155/2022/2644205 PMC8957421 35345830

[27] Chen J, Kang B, Zhao Y, Yao K, Fu C. Effects of natural dietary supplementation with Macleaya cordata extract containing sanguinarine on growth performance and gut health of early-weaned piglets. J Anim Physiol Anim Nutr (Berl). 2018;102(6):1666–74. 30129225 10.1111/jpn.12976

[28] Koopmans SJ, Guzik AC, van der Meulen J, Dekker R, Kogut J, Kerr BJ, Southern LL. Effects of supplemental L-tryptophan on serotonin, cortisol, intestinal integrity, and behavior in weanling piglets. J Anim Sci. 2006;84(4):963–71. 16543575 10.2527/2006.844963x

[29] Tossou MC, Liu H, Bai M, Chen S, Cai Y, Duraipandiyan V, Liu H, Adebowale TO, Al-Dhabi NA, Long L et al. Effect of High Dietary Tryptophan on Intestinal Morphology and Tight Junction Protein of Weaned Pig. Biomed Res Int. 2016, 2016:2912418. 10.1155/2016/2912418 PMC4913049 27366740

[30] Gershon MD, Tack J. The serotonin signaling system: from basic understanding to drug development for functional GI disorders. Gastroenterology. 2007;132(1):397–414. 17241888 10.1053/j.gastro.2006.11.002

[31] Vicentini FA, Fahlman T, Raptis SG, Wallace LE, Hirota SA, Sharkey KA. New Concepts of the Interplay Between the Gut Microbiota and the Enteric Nervous System in the Control of Motility. Adv Exp Med Biol. 2022;1383:55–69. 36587146 10.1007/978-3-031-05843-1_6

[32] DeMeo MT, Mutlu EA, Keshavarzian A, Tobin MC. Intestinal Permeation and Gastrointestinal Disease. J Clin Gastroenterol. 2002;34(4):385–96. 11907349 10.1097/00004836-200204000-00003

[33] Kojima S, Tohei A, Anzai N. A role for endogenous peptide YY in tachykinin NK(2) receptor-triggered 5-HT release from guinea pig isolated colonic mucosa. Br J Pharmacol. 2012;167(6):1362–8. 22758653 10.1111/j.1476-5381.2012.02094.x PMC3505000

[34] Laporta J, Moore SA, Weaver SR, Cronick CM, Olsen M, Prichard AP, Schnell BP, Crenshaw TD, Peñagaricano F, Bruckmaier RM, Hernandez LL. Increasing serotonin concentrations alter calcium and energy metabolism in dairy cows. J Endocrinol. 2015;226(1):43–55. 26099356 10.1530/JOE-14-0693

[35] Tang X, Xiong K, Fang R, Li M. Weaning stress and intestinal health of piglets: A review. Front Immunol. 2022;13:1042778. 36505434 10.3389/fimmu.2022.1042778 PMC9730250

[36] Čoklo M, Maslov DR, Kraljević Pavelić S. Modulation of gut microbiota in healthy rats after exposure to nutritional supplements. Gut Microbes. 2020;12(1):1–28. 32845788 10.1080/19490976.2020.1779002 PMC7524141

[37] Bervoets L, Van Hoorenbeeck K, Kortleven I, Van Noten C, Hens N, Vael C, Goossens H, Desager KN, Vankerckhoven V. Differences in gut microbiota composition between obese and lean children: a cross-sectional study. Gut Pathog. 2013;5(1):10. 23631345 10.1186/1757-4749-5-10 PMC3658928

[38] Koliada A, Syzenko G, Moseiko V, Budovska L, Puchkov K, Perederiy V, Gavalko Y, Dorofeyev A, Romanenko M, Tkach S, et al. Association between body mass index and Firmicutes/Bacteroidetes ratio in an adult Ukrainian population. BMC Microbiol. 2017;17(1):120. 28532414 10.1186/s12866-017-1027-1 PMC5440985

[39] Ushida K, Kishimoto A, Piao SJ, Itoh M, Shiga A, Nakanishi N, Tsukahara T. An epidemiological survey on pigs showing symptoms of infectious enteric diseases and dyspepsia in Japan. Anim Sci J. 2009;80(5):556–61. 20163620 10.1111/j.1740-0929.2009.00671.x

[40] Zhou J, Luo J, Yang S, Xiao Q, Wang X, Zhou Z, Xiao Y, Shi D. Different Responses of Microbiota across Intestinal Tract to Enterococcus faecium HDRsEf1 and Their Correlation with Inflammation in Weaned Piglets. Microorganisms. 2021;9(8):1767. 34442847 10.3390/microorganisms9081767 PMC8402050

[41] Pahalagedara A, Flint S, Palmer J, Brightwell G, Luo X, Li L, Gupta TB. Non-Targeted Metabolomic Profiling Identifies Metabolites with Potential Antimicrobial Activity from an Anaerobic Bacterium Closely Related to Terrisporobacter Species. Metabolites. 2023;13(2):252. 36837871 10.3390/metabo13020252 PMC9962286

[42] Chen R, Wu P, Cai Z, Fang Y, Zhou H, Lasanajak Y, Tang L, Ye L, Hou C, Zhao J. Puerariae Lobatae Radix with chuanxiong Rhizoma for treatment of cerebral ischemic stroke by remodeling gut microbiota to regulate the brain-gut barriers. J Nutr Biochem. 2019;65:101–14. 30710886 10.1016/j.jnutbio.2018.12.004

[43] Huws SA, Kim EJ, Lee MR, Scott MB, Tweed JK, Pinloche E, Wallace RJ, Scollan ND. As yet uncultured bacteria phylogenetically classified as Prevotella, Lachnospiraceae incertae sedis and unclassified Bacteroidales, Clostridiales and Ruminococcaceae may play a predominant role in ruminal biohydrogenation. Environ Microbiol. 2011;13(6):1500–12. 21418494 10.1111/j.1462-2920.2011.02452.x

[44] Wang D, Tang G, Zhao L, Wang M, Chen L, Zhao C, Liang Z, Chen J, Cao Y, Yao J. Potential roles of the rectum keystone microbiota in modulating the microbial community and growth performance in goat model. J Anim Sci Biotechnol. 2023;14(1):55. 37029437 10.1186/s40104-023-00850-3 PMC10080759

[45] Binda C, Lopetuso LR, Rizzatti G, Gibiino G, Cennamo V, Gasbarrini A, Actinobacteria. A relevant minority for the maintenance of gut homeostasis. Dig Liver Dis. 2018;50(5):421–8. 29567414 10.1016/j.dld.2018.02.012

[46] Bibbò S, Abbondio M, Sau R, Tanca A, Pira G, Errigo A, Manetti R, Pes GM, Dore MP, Uzzau S. Fecal Microbiota Signatures in Celiac Disease Patients With Poly-Autoimmunity. Front Cell Infect Microbiol. 2020;10:349. 32793511 10.3389/fcimb.2020.00349 PMC7390951

[47] Mei L, Zhou J, Su Y, Mao K, Wu J, Zhu C, He L, Cui Y. Gut microbiota composition and functional prediction in diarrhea-predominant irritable bowel syndrome. BMC Gastroenterol. 2021;21(1):105. 33663411 10.1186/s12876-021-01693-w PMC7934555

[48] Christodoulides M, de Oliveira D, Cleary DW, Humbert MV, Machado-de-Avila RA, La Ragione RM. An in silico reverse vaccinology study of Brachyspira pilosicoli, the causative organism of intestinal spirochaetosis, to identify putative vaccine candidates. Process Biochem. 2022;122:128–48.

[49] Mølbak L, Klitgaard K, Jensen TK, Fossi M, Boye M. Identification of a novel, invasive, not-yet-cultivated Treponema sp. in the large intestine of pigs by PCR amplification of the 16S rRNA gene. J Clin Microbiol. 2006;44(12):4537–40. 17005743 10.1128/JCM.01537-06 PMC1698379

[50] Zhong X, Lin P, Yao Y, Liu Z, Zhou X, Guan X, Huang J. Effects of dietary supplementation with bioactive peptides derived from rapeseed protein on the growth performance, serum biochemistry and faecal micro-organism composition of weaned piglets. J Anim Physiol Anim Nutr (Berl). 2023;107(3):867–77. 36541276 10.1111/jpn.13796

[51] Meng Q, Zhang Y, Li J, Shi B, Ma Q, Shan A. Lycopene Affects Intestinal Barrier Function and the Gut Microbiota in Weaned Piglets via Antioxidant Signaling Regulation. J Nutr. 2022;152(11):2396–408. 36774106 10.1093/jn/nxac208

